# Supplementary material for: Therapeutic hypothermia after out of hospital cardiac arrest improve 1-year survival rate for selective patients
Source: PLoS One. 2020 Jan 7;15(1):e0226956. doi: 10.1371/journal.pone.0226956 (PMC6946126; doi:10.1371/journal.pone.0226956)
Supplement: S1 Table — (DOCX) [file pone.0226956.s001.docx]

Table 1s: Inclusion and exclusion criteria

| Inclusion Criteria | Exclusion Criteria |
| --- | --- |
| 1. Age ≥18 2. Patients hospitalized in ICCU ward due to out of hospital cardiac arrest 3. Therapeutic hypothermia (only TH arm) | 1. Pregnancy 2. Do not resuscitate patient 3. Failure to achieve target cooling temperature 4. Early cessation of hypothermia from any cause 5. Patients failure to achieve return of spontaneous circulation (ROSC) 6. Cardiogenic shock on admission 7. Clear evidence of etiology as mentioned: drug abuse, head & neck trauma, active major bleeding. |
