# Supplementary material for: Therapeutic hypothermia after out of hospital cardiac arrest improve 1-year survival rate for selective patients
Source: PLoS One. 2020 Jan 7;15(1):e0226956. doi: 10.1371/journal.pone.0226956 (PMC6946126; doi:10.1371/journal.pone.0226956)
Supplement: S2 Table — (DOCX) [file pone.0226956.s002.docx]

Table 2s: Patients demographic characteristics

| p-Value | Non-Therapeutic Hypothermia  (n=35) | Therapeutic Hypothermia  (n=57) |  |
| --- | --- | --- | --- |
| .06 | 61.2±17.5  (65; 18-91) | 58.0±11.1  (56; 39-91) | Age (average ± range) |
| .07 | 23 (65.7) | 47 (82.5) | Male gender |
| .74 | 5 (14.3) | 6 (10.5) | Obesity |
| .12 | 16 (45.7) | 27 (47.4) | Smoking |
| .38 | 18 (51.4) | 24 (42.1) | Hypertension |
| .24 | 14 (40.0) | 30 (52.6) | Hyperlipidemia |
| .68 | 4 (11.4) | 5 (8.8) | Chronic renal failure |
| .73 | 8 (22.9) | 14 (24.6) | Diabetes mellitus |
| .10 | 17 (48.6) | 18 (31.6) | Ischemic heart disease |
| .10 | 7 (20.0) | 4 (7.0) | CABG |
| .07 | 8 (22.9) | 5 (8.8) | Significant Valvular disease |
| >.99 | 4 (11.5) | 8 (14.0) | Atrial fibrillation |
| .40 | 10 (28.6) | 13 (38.2) | Congestive |
