# Supplementary material for: Therapeutic hypothermia after out of hospital cardiac arrest improve 1-year survival rate for selective patients
Source: PLoS One. 2020 Jan 7;15(1):e0226956. doi: 10.1371/journal.pone.0226956 (PMC6946126; doi:10.1371/journal.pone.0226956)
Supplement: S3 Table — (DOCX) [file pone.0226956.s003.docx]

Table 3s: Cause of sepsis among study groups

| p-Value | Non-Therapeutic Hypothermia  (n=35) | Therapeutic Hypothermia  (n=57) |  |
| --- | --- | --- | --- |
| .04 | 14 (40) | 29 (50.9) | Sepsis |
|  | 8 (57.1) | 20 (69.0) | Pneumonia |
|  | 3 (21.4) | 4 (13.8) | Urinary tract infection |
|  | 1(7.14) | 1 (3.4) | Bacteremia |
|  | 5 (35.7) | 19 (65.5) | Culture positive |
