# Supplementary material for: Therapeutic hypothermia after out of hospital cardiac arrest improve 1-year survival rate for selective patients
Source: PLoS One. 2020 Jan 7;15(1):e0226956. doi: 10.1371/journal.pone.0226956 (PMC6946126; doi:10.1371/journal.pone.0226956)
Supplement: S1 Fig — (DOCX) [file pone.0226956.s004.docx]

Figure 1s: Neurological outcome - 30 days & 1-year average CPC score among study groups
