# Supplementary material for: Therapeutic hypothermia after out of hospital cardiac arrest improve 1-year survival rate for selective patients
Source: PLoS One. 2020 Jan 7;15(1):e0226956. doi: 10.1371/journal.pone.0226956 (PMC6946126; doi:10.1371/journal.pone.0226956)
Supplement: Attachment — Submitted filename: respons to reviewers 17.10.19.docx [file pone.0226956.s006.docx]

Thursday, October 17, 2019

**Dear PLOS ONE Editorial Office,**

First, I would like to thank the reviewers for their comprehensive and meticulous review and the PLOS ONE journal editors for their decision to reconsider the article for publication.

General comments:

1. The manuscript underwent extensive changes based on reviewer's comments. All changes have been highlighted in the corresponding file and labeled "revised manuscript with track changes". I will address to the specific points of the reviewers in the following paragraph. Additional unmarked version of the article will also be submitted.
2. The manuscript was professionally edited by Ediqo.com prior submission. Due to the comments of the reviewers regarding the professional editing and proofreading the manuscript was re-edit. Certification regarding professional editing will be attached to the submitted files.
3. The financial disclosure will not change
4. The manuscript has been formatted according PLOS ONE style. Tables were incorporated into text; figures were removed within the manuscript file and formatted according PACE digital diagnostics in separate files. Reference has been formatted.
5. Repository Data will be available upon acceptation. I do not have a DOI or website to upload the repository data. I will need your assistance with that.

I will address each point of the reviewers.

Reviewer #1:

1. The authors report that they included only OHCA patients in the study. Nonetheless, in table 1 it is clear that some patients suffered from cardiac arrest inside a medical facility. Please clarify this in the manuscript. The term medical facility has been changed to "Medical clinic" and description wad added to table 1. Medical clinic means a local small clinic which only offered a basic life support by professional medical staff.
2. It is not clear at what temperature TH was performed. Please clarify this in the manuscript. – Paragraph has been added to the Method section, lines 19-20
3. Please use the abbreviations that you have already defined, all over the manuscript and define the ones that have not been explained. Also, define all abbreviations used in tables and figures – Changes has been made through the entire manuscript. Definitions to Abbreviation were added to tables 1 - 4.
4. Sustained VT is not a cardiac arrest rhythm. Pulseless VT is a shockable rhythm. Please change this in the “introduction” section. We agree. It has been changed
5. Due to the grammar and language mistakes in the entire article, I strongly recommend manuscript editing by a native speaker. The manuscript has been re-edited by professional native English service company (Ediqo.com)
6. Huge part of the “introduction section” is a review of the current literature. Please move this part to the “discussion section”. – We agree. It has been changed and moved to the discussion section.
7. Clarify what “ischemic time” means and how it was measured – The term was changed to "Time to CPR" with clarification in table 1.
8. All patients were unresponsive after ROSC or did they have a good level of consciousness when they arrived at your hospital? All patients regain ROSC prior Treatment. Section 2.2. Lines 1-2 were added.
9. The patients were treated for OHCA with AEDs? How did the authors know the presenting rhythm (PEA, asystole, VF, PVT)? Please include this information in the manuscript – all of our patients were treated by EMS prior hospital arrival. Information was based on questioning of medical staff, documented monitor strip and interrogation if external defibrillator. Section 2. Methods, lines 6 to 8 were added.
10. Change sudden cardiac arrest to “cardiac arrest”. Changes have been made on entire manuscript.
11. Many statements are not followed by a reference. Please insert all necessary references. – The Reference was rearranged. Reference No. 33 was added. Reference was added to Paragraph 3 in the introduction section and to paragraph 3 in the discussion section.
12. In the Introduction section you refer to 3 categories of TH depending on the depth of cooling intensity but then you state only 2 categories. Please report all 3. – The relevant paragraph has been moved to the discussion section and was re-edited.
13. From all patients included in TH and non-TH groups you had information about the preceding symptoms, ischemic time, time for ROSC etc? If not, include the exact number of patients from whom you had information in table 1. Also state what the number in parenthesis represents. We address these points in tables 1 to 4.
14. Please define what “median time for ROSC” represents. Is it the time from the beginning of CPR by bystanders, the EMS or from the cardiac arrest? Changes have been made in table 1.

Reviewers #2

1. The issue of proper patient selection is paramount. Of note, there are published studies assessing the effect of BMI in patients who are not treated with TH and adding this information (BMI of your patients) would be important. Excellent point but unfortunately we do not have a full data on BMI score (only in small number of patients). We have documentation regarding the diagnosis of "OBESITY" but we did not found it to play a role in the outcome.
2. The non-significant benefit of TH is a result that cannot be neglected and merits further research. Notably, there was a small difference between the two groups, which could be significant if the study sample was larger. Also, your study confirms the adverse events associated with TH that have been reported by other authors and RCTs, e.g. infection, but you do not describe the method for inducing TH in your institution (initiated in ED/ICU, blankets/IV fluids, etc). Please comment. Paragraph has been added to the Method section, lines 15-20.
3. Some minor comments: Your introduction section must be organized to 3-4 smaller paragraphs. It is too large. Major part of the introduction moved and re-edited into the discussion section.
4. please add the matching process (in-detail) to the supplementary material – Description of the analysis was added to the statistical section (lines 10-12)
5. Have the manuscript reviewed by a native English speaker - The manuscript has been re-edited by professional native English service company (Ediqo.com)

Sincerely,

Dr. Koren Ofir
