# Supplementary material for: Therapeutic hypothermia after out of hospital cardiac arrest improve 1-year survival rate for selective patients
Source: PLoS One. 2020 Jan 7;15(1):e0226956. doi: 10.1371/journal.pone.0226956 (PMC6946126; doi:10.1371/journal.pone.0226956)
Supplement: Attachment — Submitted filename: Review 25.11.19.docx [file pone.0226956.s007.docx]

Tuesday, November 26, 2019

**Dear PLOS ONE Editorial Office,**

First, I would like to thank the reviewers for their comprehensive and meticulous review and the PLOS ONE journal editors for their decision to reconsider the article for publication.

I will address each point of the reviewers.

1. **In your manuscript you state that "Informed consent was not required due to the confidentiality of patent data" and also "researchers contacted the patient or close relatives for further details". If the data was kept confidential please explain how patients or their relatives were contacted** - The hospital's internal ethics committee decided there was no need for Informed consent in a retrospective study which was based mainly on information from computerized medical records. We ask the committee to allow us the opportunity to contact individual in a case were the information was not reliable or unclear. In this case the committee did conditional the use of information with the patient's approval. In our study, eventually no contact was made. The information obtained from the computerized systems of the hospitals and the HMO was reliable. The paragraph regrading it in the Methods section was changed.
2. **In your Methods section, please provide additional details regarding your statistical analyses. A sample size calculation is referred to but not described, nor an appropriate sample size stated. Also, no post-hoc corrections for multiple comparisons are mentioned in your manuscript. If these were performed please include them in the text, or justify their absenc. –** Methods and Statics section has been changed.
3. **Please revise parts of your manuscript (and title) that use the phrase "proper patient selection". This is unclear and open to misinterpretation in the context of whether adequate clinical practice was followed for the patients included in the study, can you therefore revise the title and references to this in the text to ensure it aligns to the analysis undertaken as part of the study** – We rephrase the title. We hope this formulation will encourage curiosity in reading the article and will not attract unnecessary criticism. We would greatly appreciate suggestions from the editorial team for a different title if the suggested title does not match.
4. **Additionally, avoid referring to any "causal" links in the text. The retrospective nature of this study does not allow for these to be made, please revise references to ‘effectiveness’ or ‘efficacy’ and tone down your 'Conclusions' section accordingly**. – The conclusion section has been changed.

Sincerely,

Dr. Koren Ofir
